# Use of the sludge obtained from the electrocoagulation process of pumping waters of fishmeal factories for feeding *Tenebrio molitor* larvae

**DOI:** 10.1016/j.heliyon.2023.e16200

**Published:** 2023-05-12

**Authors:** Edwar Aguilar-Ascón, Daniel Pariona-Velarde, Raúl Loayza-Muro, Miguel Albrecht-Ruíz

**Affiliations:** a Universidad de Lima, Instituto de Investigación Científica, Grupo de Investigación en Tecnologías Exponenciales, Estudios Generales, Av. Javier Prado 4600, Surco, Lima, Peru; b Laboratorio de Bioquímica, Dirección de Investigación (DIDITT), Instituto Tecnológico de la Producción, Carretera a Ventanilla Km. 5, 2, Callao, Peru; c Laboratorio de Ecotoxicología, Facultad de Ciencias y Filosofía, Universidad Peruana Cayetano Heredia, Av. Honorio Delgado 430, San Martín de Porres, 15102, Peru

**Keywords:** *Tenebrio molitor* larvae, Electrocoagulation of pumping water, Feeding with sludge from electrocoagulation

## Abstract

Sludge residue from pumping water treatment obtained by electrocoagulation process (LEC) in fishmeal factories, was used as a feeding ingredient for *Tenebrio molitor* larvae. LEC was conditioned by three bioprocesses: fermentation with Lactobacillus casei, fermentation with Sacharomyces, and hydrolysis with pancreatin enzymatic mixture. Soybean isolate was used as a control. Larvae consuming LEC-containing diets presented a higher weight gain rate than the controls. The proximal larvae dry basis composition values of fat, ash, and protein (37.2% ± 2%, 3.9% ± 0.4%, and 50.2% ± 4.9%, respectively) did not present significant intergroup differences. LEC contained 4.2% aluminum and its conditioning through fermentation with lactic bacteria reduced its bioavailability in the larvae, with values similar to those of controls (3.9 ± 0.7 μg Al/g). The iron content in LEC-fed larvae was higher than that in the control group, while their fatty acid profile was only slightly different. These initial results with LEC, which organic material is difficult to hydrate and assimilate, suggest its suitability as a protein source and attractant for a faster growth of *T. molitor* larvae.

## 1 Introduction

The increases in organic waste as well as its management are one of the main environmental concerns worldwide. The fishing industry increasingly tends to contaminate marine waters, making it necessary to find alternatives to reduce its impacts [1]. During the last decade, fishmeal manufacturing in Peru has presented an average annual landing of 4.21 million tons of anchovy (*Engraulis ringens*) [2]; where large volumes of water transporting fish from warehouses to factories is contaminated. This is called “pumping water” and contains suspended solids with a high load of contaminants [3]. To reduce the organic load and comply with health-environmental regulations, the “pumping water” is currently treated with coagulants and flocculants before returning to the sea. This treatment generates a residual sludge containing oxidized organic matter and high iron content.

The electrocoagulation process is an alternative method for treating contaminated water where an electric current is applied to immersed electrodes to remove organic and inorganic solids [4,5]. Its high effectiveness, low maintenance cost, less workforce, and less use of chemicals makes it a promising treatment method. The most commonly used electrodes are those made of aluminum and iron since they are readily available, cheap, and effective. Moreover, this system is effective for removing suspended organic solids, as well as dissolved metals. Recent investigations have applied this technology to “pumping water” from the anchovy fishmeal industry using aluminum electrodes [6], allowing the recovery of 99% of total solids as sludge. These contained 1.35%–2.59% of aluminum from the electrochemical wear of electrodes, 0.22%–0.37% of iron, and 1.9%–2.65% of phosphorus.

The management of this sludge would imply its disposal in landfills and consequent environmental impacts. An alternative solution would be to use LEC as a feed ingredient. Although its high salt content and insolubility makes it unsuitable for direct use in biological systems, bioconversion processes by fermentation would be an alternative solution for its subsequent use as feed ingredient. Population growth has increased the global demand for food, especially for animal protein sources, intensifying the production of highly resource efficient animal feed, such as fishmeal. However, as the population growth rate continues to increase, protein sources will have to expand by finding less-used alternatives [7]. Insect farming is among the options to address food security. Due to their rapid reproduction and high growth and conversion rates, insects can be reared anywhere using diverse residues under controlled conditions, with low environmental impacts [8]. Insects have protein, fat, and minerals levels similar to those of meat products, and can be eaten whole, ground, or incorporated into other foods. Large-scale use of insects as an ingredient in the composition of feed is technically feasible, and companies in several parts of the world are using them in aquaculture and poultry feed. The black soldier fly (*Hermetia illucens*) larvae and the flour beetle (*T. molitor*) larvae are among the most used insects in animal feed. These larvae are primary consumers of organic waste which ingestion and digestion trigger physiological and biochemical events that lead to a high conversion of waste into proteins, carbohydrates and lipids [9].

*T. molitor* (Tenebrionidae) has an endemic worldwide distribution, and is considered a secondary pest. Its mature larvae are light yellow colored, 20–32 mm long, 130–160 mg weight, and are used as live food for reptiles, birds, and fish [10]. The use of Tenebrio larvae has some advantages, such as the requirement of less space for rearing, their ability to consume different lignocellulosic residues, and growth at low relative humidity. They are omnivorous, can eat and bioconvert all types of organic materials, and can be used for animal feed, even replacing fishmeal [9,11,12]. Another advantage over animal proteins is the lower generation of enteric methane per gram of protein produced and lower water consumption [13].

The use of treated LEC by fermentation and hydrolysis to increase its bioavailability as a supply of feed for *Tenebrio* larvae could be an alternative for waste management in the fishmeal industry. Hence, this study aimed at the bioconversion of LEC to explore its potential use as a feed ingredient for *T. molitor* larvae. To achieve this goal, the electrocoagulation sludge was dried and conditioned, and included as a protein source in the larvae diet.

## 2 Materials and methods

### 2.1 Rearing

**Fig. 1:**
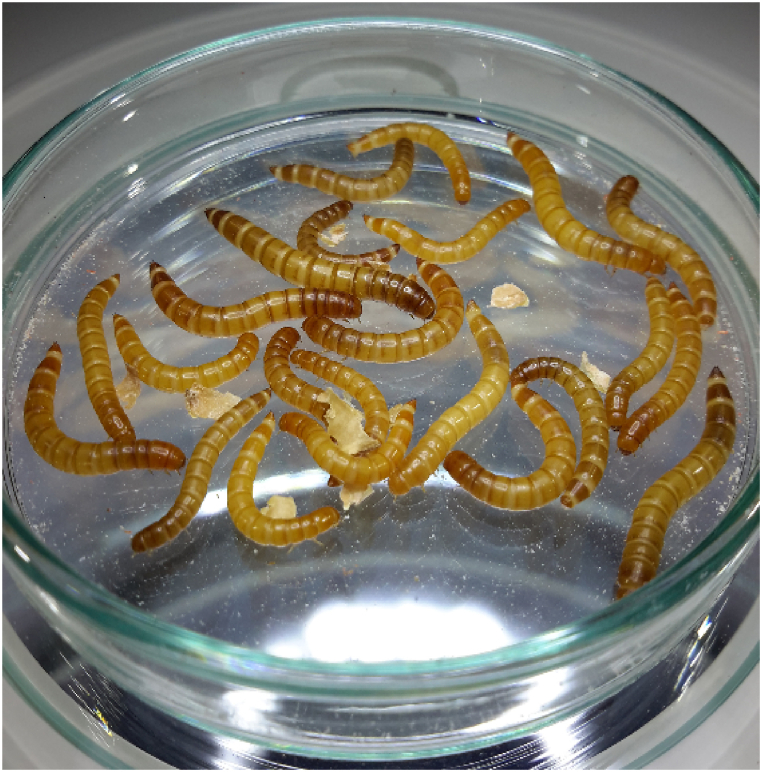
*Tenebrio molitor* larvae.

*T. molitor* larvae (Fig. 1) were reared in the laboratory under controlled relative humidity (50%–70%) and temperature (20°C-25 °C) conditions, fed with a diet based on wheat fiber (60%), starch (20%), soy isolate (20%), Fleischmann® Instant Yeast (1%), and 431® Chr Hansen *Lactobacillus paracasei* (1%) added as B complex supplement. Sludge obtained from pumping water electrocoagulation process (LEC) was dried and transformed into meal (EC-meal). The latter was used as raw material for the fermentation or hydrolysis processes aimed to feed Tenebrio larvae.

**Fig. 2:**
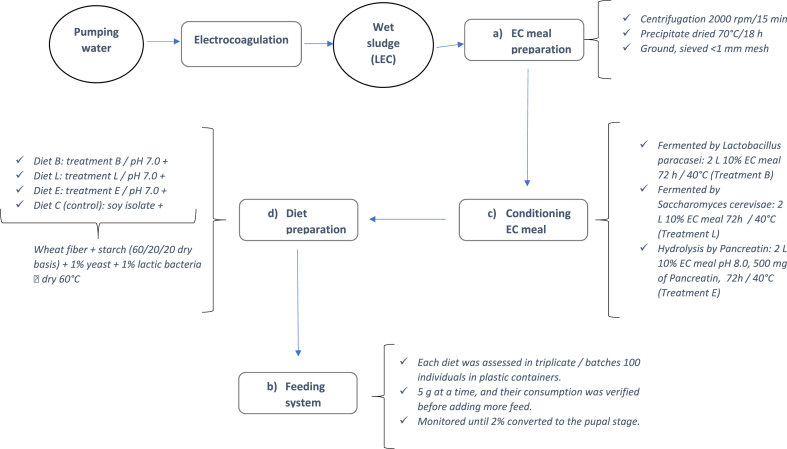
Sequence for preparing diets and feeding larvae.

Diets for Larvae (Fig. 2):

a) Preparation of the “EC meal”: LEC was collected from the coagulation tank and centrifuged at 2000 rpm for 15 min to separate the liquid phase. The precipitates were then placed on trays and dried at 70 °C for 18 h in a convection oven. The dry sample was ground and sieved using a <1 mm mesh. The “EC meal” was stored in polyethylene bags until use.
b) Conditioning of the “EC meal” through fermentation or hydrolysis: The “EC meal” was fermented or hydrolyzed to promote bioconversion. Fermentation conditioning was performed with *Lactobacillus paracasei* 431® (code B) or with the brewer yeast *Saccharomyces cerevisae* var. Bayanus Maurivin PDM® (code L). A total of 5 g of “EC meal” and 1 g of sucrose were added to 100 mL of distilled water to adapt the bacteria or yeast to the protein substrate. The pH was adjusted to 8.0 and 1 g of lactic acid bacteria or 1 g of dry yeast were added. The cultures were incubated for 24 h at 40 °C with constant agitation, and the pH was adjusted every 4 h. After the adaptation period, the mixture was centrifuged and the sediment was added to a 10% “EC meal” solution in a final volume of 2 L and then incubated for 72 h at 40 °C with constant agitation. The pH was adjusted every 4 h to avoid acidification.

The enzyme blend Pancreatin (code E), obtained from porcine pancreas (350 FIP-U/g Protease, 6000 FIP-U/g Lipase, 7500 FIP-U/g Amylase) was used for conditioning through hydrolysis. A total of 15 g of “EC meal” was placed in 2 L of water, the pH was adjusted to 8.0, and 500 mg of Pancreatin was added. The mixture was incubated and treated under the same conditions used in the fermentation process.

c) Diet preparation: A mixture of wheat, soy isolate, and starch in a proportion of 60/20/20, respectively, was used as the control diet (code C), which was supplemented with 1% dry yeast (Maurivin PDM®) and 1% lyophilized lactic bacteria (*L. paracasei*-431®). After conditioning, the pH of the diets containing “EC meal” was adjusted to 7.0, and wheat fiber and starch were added in sufficient quantity to maintain a 60/20/20 ratio on a dry basis, similar to that of the control. This mass was dehydrated at 60 °C after mixing until reaching 15% moisture; the sample was then homogenized in a grinder, packed in vacuumed bags, and kept refrigerated until use.
d) Feeding system: Each diet was assessed in triplicate, in batches containing 100 individuals placed in plastic containers. The diets were discreetly added to the containers, 5 g at a time, and their consumption was verified before adding more feed. A total of 5 g of fresh carrot slices were added every 2 weeks as a supplement of moisture, vitamins, and minerals.
e) Growth record of larvae: Growth was monitored until 2% of the larvae had reached the pupal stage and was evaluated by measuring the initial and final weight with an analytical balance.

### 2.2 Physical-chemical tests

The proximal composition of the samples was determined following methodologies recommended by the FAO [14]. Moisture content was determined by drying at 104 °C until reaching constant weight, ash content by muffle calcination at 600 °C for 12 h, crude fat was quantified in a Soxtherm® device by continuous extraction with hexane, and protein was obtained using the Kjeldahl method using the kjeldatherm/Turbosog/Vapodest® equipment system (C. Gerhardt GmbH & Co. KG Cäsariusstraße 97, 53639 Königswinter. Germany) and multiplying the nitrogen content by 6.25.

Iron (Fe) was quantified using atomic absorption spectrometry (AAS - PerkinElmer AAnalyst 800) following the 999.11 AOAC methodology [15], and phosphorus content (P) was determined using the photometric technique 965.17 AOAC using a Thermo Fisher Genesys 180 Spectrophotometer. For aluminum (Al) quantification the sample was calcined at 600 °C, dissolved in 1 N HCl, and analyzed by AA at 396.2 nm, using a nitrous oxide-acetylene flame and following the equipment manufacturer’s recommendations [16].

The fatty acid profile was determined in the fat extracted from the larvae using the Blight & Dyer methodology [17]. The methylated fatty acids [18] were separated using gas chromatography with a flame ionization detector (PerkinElmer Autosystem XL GC System): Injection 250 °C/Detector 270 °C, oven initial temperature 170 °C, final temperature 220 °C, rate 1 °C/min, SUPELCOWAX™ 10 column (L × I.D. 30 m × 0.32 mm, 0.25 μm coating).

### 2.3 Statistical analysis

Preliminary analyses were conducted to confirm the assumptions of normality, linearity, and homoscedasticity. The final weights and the growth ratios and chemical composition (metals, fatty acids, and proximal chemical composition) at the end of the rearing period were compared through an analysis of variance (p < 0.05) using the Statgraphics Centurion XV software.

## 3 Results and discussion

The dry sludge obtained from pumping water electrocoagulation (“EC meal”) presented a rancid odor and very low solubility in water. The proximal composition (on a dry basis) was 36.9% protein, 25.3% fat, and 25.0% ash, containing 4.2% aluminum, 278 mg/kg iron, and 2.6% phosphorus (Table 1a). Its fatty acid profile shows that the levels of w-3 polyunsaturated fatty acids (EPA and DHA) were well below those reported for anchovies [19], confirming the rancidity of the fat in the sludge obtained by electrocoagulation (Table 1b).

**Table 1a:** Proximal composition, aluminum (%), phosphorous (%) and iron (mg/kg) content (dry basis) in “EC meal”.

Composition	%
Protein	36.9 ± 2.3
Fat	25.3 ± 0.6
Ash	25.0 ± 2.5
Not quantified	12.7 ± 5.2
Al	4.2 ± 0.2
P	2.6 ± 0.2
Fe (mg/kg): 277.6 ± 16

**Table 1b:** Fatty acid profile (%) from “EC meal” fat extracted.

Fatty acids	%
C 14:0 (Myristic)	10.7
C 16:0 (Palmitic)	30.3
C 16:1 (Palmitoleic)	9.1
C 18:0 (Stearic)	7.4
C 18:1 w-9 (Oleic)	9.5
C 18:1 w-7 (Vaccenic)	4.4
C 18:2 w-6 (Linoleic)	1,0
C 20:5 w-3 (Eicosapentaenoic)	4.4
C 22:5 w-3 (Clupadonic)	1.7
C 22:6 w-3 (Docosahexaenoic)	8.1
Total	86.6
∑ W-3	14.2

The “EC meal” was conditioned via fermentation or hydrolysis before preparing the diets to increase nutrients bioavailability. Therefore, part of the proteins, lipids and low molecular weight molecules responsible for the rancid odor would be metabolized during conditioning by fermentation, and the *de novo* synthesized molecules could chelate metals catalyzing oxidation, such as Fe or Al [[20], [21], [22], [23]]. For conditioning through hydrolysis, it was assumed that Pancreatin (a mixture of proteases, lipases and amylases) would hydrolyze the biomolecules of the “EC meal”, facilitating their assimilation by the larvae.

Wheat bran was used as a fiber source in all the diets and in the control group, and soy isolate was used as a protein source (diet C). The “EC meal” containing diets were classified after conditioning by fermentation with lactic acid bacteria (diet B), brewer's yeast (diet L), or hydrolysis (diet E). Each type of conditioning generated different bioavailability, which was reflected in the growth time, weight, and proximal composition of the larvae. Dried brewer's yeast (1%) and *L. casei* (1%) were added to all the prepared diets to improve nutrient absorption and to increase growth factors and tolerance to high mineral load.

### 3.1 Larval growth

The diets prepared from the conditioned “EC meal” (diets B, L, and E) improved larvae acceptance compared to the control group, showing an increase of almost twice the weight gain ratio observed in diet C (Table 2). Moreover, there were no significant differences between diets B, L, and E.

**Table 2:** Weight ratios throughout the growth of *Tenebrio molitor* larvae.

DIET	Initial weight (g)	Final weight (g)	Initial weight ratio	Final weight ratio	Larval weight (mg/days)	Weight gain ratio
Control (**C**)	31.61 ± 0.98	71.1 ± 1.21^b^	1.00 ± 0.00^a^	1.00 ± 0.00^a^	0.42 ± 0.08^a^	1.25 ± 0.12^a^
Fermentation with lactic bacteria (**B**)		98.5 ± 0.47^a^	0.95 ± 0.04^a^	1.40 ± 0.05^b^	0.69 ± 0.04^b^	2.12 ± 0.11^b^
Yeast fermentation (**L**)		102.5 ± 3.24^a^	1.02 ± 0.05^a^	1.46 ± 0.05^b^	0.72 ± 0.10^b^	2.24 ± 0.11^b^
Enzymatic hydrolysis (**E**)		96.6 ± 1.80^a^	0.97 ± 0.06^a^	1.45 ± 0.07^b^	0.72 ± 0.05^b^	2.06 ± 0.20^b^

Different letters in the same column indicate significant differences (p < 0.05).

The metabolism of the larvae allows them to feed on a wide variety of food and to adapt to different matrices maintaining the ratio of their chemical composition (Table 3). Changes in the immune and reproductive systems of the larvae could have occurred as a compensatory response, although this was not assessed in this study [24].

**Table 3:** Proximal composition (%) of *Tenebrio molitor* larvae after feeding on diets C, B, L and E.

WET BASIS (%)					DRY BASIS (%)		
Code	Moisture	Protein	Fat	Ash	Protein	Fat	Ash
Control (**C**)	59.4 ± 3.5	20.8 ± 0.9	14.0 ± 0.0	1.6 ± 0.2	56.2 ± 2.5	38.0 ± 0.0	4.4 ± 0.5
Fermentation with lactic bacteria (**B**)	58.7 ± 1.7	20.6 ± 0.4	14.3 ± 0.0	1.5 ± 0.1	50.0 ± 1.0	34.6 ± 1.4	3.6 ± 0.1
Yeast fermentation (**L**)	58.6 ± 2.2	18.5 ± 1.8	14.8 ± 0.8	1.5 ± 0.1	47.2 ± 4.5	37.9 ± 1.5	3.7 ± 0.2
Enzymatic hydrolysis (**E**)	58.8 ± 1.5	18.7 ± 1.9	15.1 ± 1.2	1.6 ± 0.1	47.2 ± 4.9	38.2 ± 2.2	3.9 ± 0.2

One of the main characteristics of the “EC meal” was its rancid aroma, similar to that of very rancid fishmeal, which would make it unviable for many species. However, the low molecular weight volatile and soluble compounds, responsible for these characteristics, could be considered food attractants for other species. In this sense, it has been described that the beetle *Tribolium castenum* prefers to feed on rancid flours due to the presence of fungi in cotton seeds and wheat bran [25]. This behavior arises from its natural development cycle; the larvae are endemic to warehouses, where they feed on flour residues in different stages of decomposition. The larvae odoriferous and gustatory sensory systems, necessary to locate food, are a heritable characteristic allowing them to adapt to different conditions. This study has taken advantage of these systems by inducing a higher diet consumption and thus developing a potentially attractive food for larvae from an otherwise stale product.

### 3.2 Proximal composition of the larvae

The proximal composition values of diets C, B, L, and E did not present significant differences. Nonetheless, it is worth noting that the larvae on diet C presented higher protein content than those fed with other diets (Table 3). In this regard, changes in the proximal composition of *Tenebrio* have only been observed on diets with a severe restriction in some of its components, or under extreme changes in growth conditions, thus disrupting their wide range of homeostasis [13,24].

The lack of significant differences between all treatments could indicate that the bioavailability of carbohydrates, protein, lipids, and minerals in the diets was equivalent. Repulsion to food would decrease its intake and, consequently, the protein and fat content. However, this did not occur during the study; probably, the bioprocesses generated during the conditioning of the “EC meal” favored the use of fats, proteins, and minerals, considering that the diets were isocaloric for carbohydrates.

Not finding differences implies that the treatments were equivalent, or that the larvae could adapt to different diets. Therefore, treated electrocoagulation residues, represent a bioavailable source of protein and fat, that helped growth as much as the control diet (with soy as a protein source). Indeed, the weight gain ratio was higher for treatments than for controls. We could not find evidences on the use of sludges from electrocoagulation as a diet for any organism; however, the use of lipids and proteins in this residue by the larvae reinforces the importance and novelty of this work. Biotransformations made by enzymes or micro-organisms on electrocoagulation sludges may have increased its assimilation and reduced metallotoxicity.

The protein content in the different diets varied from 47.2% to 56.2% (dry basis), which is slightly higher than that reported by Ref. [13] (46.9%–48.6%) in different types of diets. However, these protein values have been overestimated, because there is a high amount of non-protein nitrogen (between 11% and 26%), mainly as a constituent of chitin [26]. The crude fat content obtained from the different diets ranged from 34.6% to 38.2%, which was also higher compared to that reported by Ref. [13] (26.3%–27.6%), decreasing to 18.9% when the caloric intake was lower. The lipid and protein content obtained in our study were higher than those reported in the literature despite the high content of Al in the “EC meal” used in the diets.

The unquantified difference in proximal composition is mainly related to the chitin in the larval cuticle. Therefore, feeding *Tenebrio* with the sludge obtained from electrocoagulation, in addition to being a source of fat and protein for animal feed, would also be an alternative pathway for the production of chitin, a potentially useful product.

### 3.3 Concentration of minerals in the larvae

The minerals could be assimilated or excreted after food biodegradation by the larvae. To avoid overestimating the metal content, once the larvae had completed their growth, they were left without food for 16 h so they could finish defecating and eliminating the non-assimilated elements. Then, they were processed and analyzed (Table 4).

**Table 4:** Aluminum, iron, and phosphorus content (dry basis) in *Tenebrio* larvae after feeding on diets C, B, L and E.

DIET	Al (ug/g)	Fe (ug/g)	P (mg/100 g)
Control (**C**)	16.5 ± 1.2^a,b^	2.4 ± 0.5^a^	1069 ± 62^a^
Fermentation with lactic bacteria (**B**)	9.6 ± 3.3^a^	17.3 ± 2.9 ^b^	779 ± 17 ^b^
Yeast fermentation (**L**)	89.6 ± 70.2^c^	14.3 ± 9.1^a,b^	843 ± 32 ^b^
Enzymatic hydrolysis (**E**)	33.5 ± 17.9 ^b,c^	38.3 ± 8.2^c^	917 ± 227^a,b^

Different letters in the same column indicate significant differences (p < 0.05).

The implementation of the electrocoagulation process is mainly aimed at water purification, and not at the collection and use of the sludge. During electrocoagulation, the wearing observed on the Al anode forms the coagulants that trap the suspended solids. This Al can be cytotoxic if used in feed for farm animals, and therefore an appropriate option would be to use it as food for *Hermetia* or *Tenebrio-type* insects, which can bioprocess waste with high content of metals, metalloids, and even heavy metals. A study on *Tenebrio* and black soldier flies exposed to heavy metals (Cd, Pb, and As) has shown bioaccumulation patterns that were significantly different for each element [27], suggesting that metal uptake may not be tightly regulated in insects. After absorption, these elements can bind to metallothioneins capable of chelating metals and preventing cell damage. They can also be captured by the microbiota in the digestive tract or in vesicles and excreted through exocytosis.

In addition, the control diet presented higher phosphorus content than those with the conditioned “EC meal”. This difference could be related to the phospholipid content, which could lead to a higher muscle mass. In this sense, the phosphorus levels in the larvae have been associated with different muscle indexes, phospholipid levels, and phosphorylation states [28]. The control diet provided a higher bioavailability of phosphorus, which could be assimilated in the form of phospholipids. Another possible cause for these differences could be that the metabolites found in the fermented and hydrolyzed “EC meal” may cause stress and increase the lipolytic activity of the larvae, decreasing the phospholipid concentration.

A concentration of 0.9 μg/g Fe was achieved in larvae fed with the control diet, although the supplied substrates did not have a significant contribution. Other studies [12,22] show that the iron values in *Tenebrio* were equivalent to those of meat (approximately 70 μg/g). Furthermore, in this study, the diets were not only supplemented with dry yeast, but also with lactic bacteria, which could modify Fe absorption in the gastrointestinal tract.

An inverse relationship was determined between phosphorus and Fe, with a correlation coefficient of 0.98 (p < 0.05). The assimilation of these elements may be determined by the presence of phosphates. Absorption is a function of the system’s Fe/P ratio, as well as the biotransformation by fermenting microorganisms or the modification of its bioavailability through enzymatic action [22].

The “EC meal” had relatively higher Fe content than the soy protein. If this were only related to the presence of these components, the diets would have had similar availability for the larvae. However, it was observed that the Fe content in the larvae was significantly different between the diets (Table 4). Therefore, the conditioning processes of the “EC meal” always generated an increase in Fe absorption, whether it was through fermentation or hydrolysis. The highest bioavailability was observed in diet E (“EC meal” conditioned by hydrolysis) followed by diet B (fermented with lactic acid bacteria) and diet L (fermented with yeast). The increase in Fe absorption in diet E would be explained by the fact that the lipase and proteolytic activity of pancreatin releases Fe, making it more bioavailable (Table 4). It is important to note that diet pH had to be adjusted before the drying process to avoid differences in Fe solubility.

The fermenting microorganisms that participated in the conditioning of the “EC meal” both in diets B and L slightly increased the bioavailability of Fe compared with the control diet. In this sense, it has been reported in studies on lactic fermentation of flour doughs for bread supplemented with ferrous sulfate that Fe bioavailability could be increased by high levels of ferritins, regardless of whether this depended on organic or inorganic Fe [22,29]. This is relevant in our case because Fe in diets B and L may associate with proteins from the lactic bacteria and yeasts, thus increasing its absorption.

Hydrolyzed proteins have a high capacity for chelating metals, including Fe [29]. Not only would the peptides chelate, but also the amino acids released, thus improving the bioavailability of inorganic Fe. Due to proteolysis, the metals associated with proteins were likely released by the electrocoagulation process, thus increasing their bioavailability to interact with the peptides and amino acids generated.

### 3.4 Fatty acid profile in the larvae

The larvae maintained a similar fatty acid profile in all the investigated diets, mainly in their content of oleic, linoleic, and palmitic acids (Table 5). The values were similar to others reported for other beetles and larvae fed with wheat bran or other sources of cellulose [[30], [31], [32], [33]]. However, it should be noted that the profile of the larvae fed with diets B, L, and E presented slightly significant differences from the control diet, such as lower content of palmitoleic acid (C16:1), linoleic acid (C18:3 w-3), and alpha-linolenic acid (C18:2 w-6), and higher content of stearic acid (C18:0) and oleic acid (C18:1w-9). In addition, no significant differences between the larvae fed with diets B, L, and E were observed.

**Table 5:** Profile of fatty acids (%) in *Tenebrio molitor* larvae after feeding on diets C, B, L and E.

Fatty acids (%)	DIET C	DIET B	DIET L	DIET E
C 14:0 (Myristic)	4.09 ± 0.18	4.43 ± 0.05	4.74 ± 0.26	4.41 ± 0.03
C 16:0 (Palmitic)	16.71 ± 0.87	18.38 ± 0.36	18.11 ± 2.35	17.51 ± 0.53
C 16:1 (Palmitoleic)	3.25 ± 0.19^b^	2.49 ± 0.12^a^	2.64 ± 0.09^a^	2.62 ± 0.12^a^
C 18:0 (Stearic)	2.21 ± 0.40^b^	2.96 ± 0.11^a^	2.91 ± 0.23^a^	2.59 ± 0.08^b^
C 18:1 w-9 (Oleic)	43.87 ± 0.50^b^	48.9 ± 0.22^a^	48.08 ± 2.64^a^	48.78 ± 0.75^a^
C 18:2 w-6 (Linoleic)	26.26 ± 0.32^b^	19.54 ± 0.27^a^	19.61 ± 0.38^a^	20.71 ± 0.30^a^
C 18:3 w-3 (a-Linolenic)	0.75 ± 0.01^b^	0.38 ± 0.01^a^	0.39 ± 0.03^a^	0.40 ± 0.01^a^

Different letters in the same row indicate significant differences (p < 0.05).

Even though the sludge obtained in electrocoagulation presented a profile of fatty acids with a percentage of EPA of 4.5% and DHA of 8.0% (Table 1a, Table 1ba and 1b), these were not present in the larvae as they were metabolized by them or during the meal conditioning processes. The w-3 fatty acids are long-chain highly unsaturated acids and thus very susceptible to oxidation. It seems that the oxidized fats could be used by the larvae, hence maintaining homeostasis.

## 4 Conclusions

The sludge from electrocoagulation (LEC) can be used as a feed ingredient for *T. molitor* larvae. It constitutes a source of proteins and food-attracting compounds that lead to a faster growth when provided in the diet. The oxidized fat content in the LEC did not prevent the larvae from consuming it. Moreover, the larvae fed on diets that included LEC presented a higher weight gain ratio during their growth; however, their proximal composition was like that of the control diet. LEC presented high concentrations of Al, but its absorption could be modulated by the type of conditioning. Thus, if the *Tenebrio* larvae were to be used to feed breeding animals, an option would be to ferment the sludge with lactic bacteria to reduce the absorption of Al. Therefore, the use of LEC as an input for the massive production of these larvae would be an option to consider.

This study shows that fermentation improves the bioavailability of the electrocoagulation sludge (LEC), coming from water depuration of the fishing industry, for feeding Tenebrio larvae, which can metabolize these residues regardless of Al or Fe content. This may provide industrial alternatives that generate profits and reduce water and environmental contamination.

This study shows that fermentation improves the bioavailability of the sludge from electrocoagulation (LEC) and can feed *Tenebrio* larvae. Hence, the residues from the water depuration of the fishing industry can be used as a protein source for rearing *Tenebrio* larvae, which can metabolize these residues regardless of the Al or Fe content; concatenating both processes in a potential circular economy scheme.

## Funding statement

To the Instituto de Investigación Científica (IDIC) of the Universidad de Lima, Pesquera Diamante S. A., Instituto Tecnológico de la Producción (ITP) and the Programa Nacional de Innovación en Pesca y Acuicultura (PNIPA)-PESSIA-PP-000001, Contract: No. 141–2018, for fully supporting the development of this study.

## Author contribution statement

Edwar Aguilar-Ascón: Conceived and designed the experiments; Contributed reagents, materials; Wrote the paper.

Daniel Pariona-Velarde: Performed the experiments; Analyzed and interpreted the data; Wrote the paper.

Raúl Loayza-Muro: Contributed reagents, materials; analysis tools or data; Wrote the paper.

Miguel Albrecht-Ruíz: Conceived and designed the experiments; Contributed reagents, materials, analysis tools or data; Wrote the paper.

## Data availability statement

Data included in article/supplementary material/referenced in article.

## Declaration of competing interest

The authors declare that they have no known competing financial interests or personal relationships that could have appeared to influence the work reported in this paper
